# Correction: Molecular dynamics simulation of the mechanical properties of multilayer graphene oxide nanosheets

**DOI:** 10.1039/c8ra90051e

**Published:** 2018-06-21

**Authors:** Xu Zhang, Shuyan Liu, Hang Liu, Jinwen Zhang, Xiaoning Yang

**Affiliations:** State Key Laboratory of Material-Oriented Chemical Engineering, College of Chemical Engineering, Nanjing Tech University Nanjing 210009 China Yangxia@njtech.edu.cn; Apparel, Merchandising, Design and Textiles Composite Materials and Engineering Center, Washington State University Pullman WA 99164 USA; School of Mechanical and Materials Engineering, Composite Materials and Engineering Center, Washington State University Pullman WA 99164 USA

## Abstract

Correction for ‘Molecular dynamics simulation of the mechanical properties of multilayer graphene oxide nanosheets’ by Xu Zhang *et al.*, *RSC Adv.*, 2017, **7**, 55005–55011.

The authors regret that Hang Liu’s name was spelled incorrectly in the original article. The correct author names are as presented above.

The Royal Society of Chemistry apologises for these errors and any consequent inconvenience to authors and readers.

## Supplementary Material

